# Seroprevalence of Brucellosis in Buffalo Worldwide and Associated Risk Factors: A Systematic Review and Meta-Analysis

**DOI:** 10.3389/fvets.2021.649252

**Published:** 2021-06-04

**Authors:** Jun-Feng Shi, Qing-Long Gong, Bo Zhao, Bao-Yi Ma, Zi-Yang Chen, Yang Yang, Yu-Han Sun, Qi Wang, Xue Leng, Ying Zong, Jian-Ming Li, Rui Du

**Affiliations:** ^1^College of Chinese Medicine Materials, Jilin Agricultural University, Changchun, China; ^2^College of Animal Science and Technology, Jilin Agricultural University, Changchun, China; ^3^Laboratory of Production and Product Application of Sika Deer of Jilin, Jilin Agricultural University, Changchun, China; ^4^Key Lab of Animal Production, Product Quality and Security, Ministry of Education, Jilin Agricultural University, Changchun, China

**Keywords:** brucellosis, *Brucella*, buffalo, meta-analysis, seroprevalence

## Abstract

**Background:** Brucellosis is an important zoonotic disease caused by *Brucella* spp. Brucellosis is widely distributed in more than 160 or 170 countries around the world, where it poses a huge threat to animal husbandry and human health. About 150 million head of water buffalo, distributed across more than 40 countries worldwide, are kept for the purposes of service, milk, and meat. High incidence of *Brucella* spp. in buffalo has negatively affected dairy products and meat products.

**Results:** We searched all research related to seroprevalence of brucellosis in water buffalo anywhere in the world in PubMed, Science Direct, SpringerLink, China National Knowledge Infrastructure, Wanfang Database, and VIP Chinese Journal Databases. A total of 26 articles published from 1985 to 2020 met the final selection criteria. The overall seroprevalence of buffalo brucellosis worldwide was 9.7%. The seroprevalence before 2010 (20.8%) (95% CI: 5.6–42.2) was much higher than the seroprevalence rate from 2010 to 2020 (4.2%) (95% CI: 1.8–7.5). Subgroup analysis by feeding mode found that the point estimate of seroprevalence in stock buffalo (11.5%) (95% CI: 3.6–23.0) was higher than that in captive buffalo (10.6%) (95% CI: 4.9–18.1). Subgroup analysis by farming mode found that the seroprevalence was higher in captive-bred buffalo (10.7%) (95% CI: 6.6–15.7) than in intensively farmed buffalo (8.5) (95% CI: 0.9–22.2). The seroprevalence in buffalo living in dry lands (6.4%) (95% CI: 2.0–12.9) is greater than that in buffalo living in wetlands (5.1%) (95% CI: 1.8–10.4) (*P* < 0.05). The seroprevalence in female buffalo (10.1%) (95% CI: 3.4–19.7) was higher than that in male buffalo (4.4%) (95% CI: 2.0–7.4). The seroprevalence in lactating buffalo was higher than that in buffalo of other ages (26.9%) (95% CI: 1.8–66.5). Subgroup analysis by detection method found that the seroprevalence detected by the complement fixation test (27.3%) (95% CI: 0.7–70.8) was much higher than that detected by other methods.

**Conclusion:** The results of this meta-analysis showed that buffalo brucellosis infection is very common in buffalo herds around the world. Although the seroprevalence of brucellosis in buffalo and humans is relatively low, serious effects upon animal husbandry and public health make it necessary to take effective control and preventive measures to control the spread of this disease.

## Introduction

Brucellosis, also known as Mediterranean fever or Malta fever (1), is one of the most common zoonotic diseases worldwide, endemic in more than 170 countries and regions (2–4). Classical taxonomists recognize six species of the causative bacterium, *Brucella*, based on subtle phenotypic and antigenic differences and host specificity: *B. abortus* (bovine), *B. melitensis* (caprine and ovine), *B. ovis* (ovine), *B. canis* (canine), and *B. neotomae* (desert wood rat) (5); some of these *Brucella* spp. are classically divided into biovars. Brucellosis can affect phagocytes, mainly infecting the reproductive system and lymph nodes. Its typical clinical presentation in female animals includes abortion of the calf, and in male animals, orchitis, epididymitis, and arthritis (6, 7). The highest infection rate among wild animals is found in American bison and bison (39.9%), followed by alpine bison and goats (33%). Water buffalo, a common domestic animal, is also a major locus of brucellosis infection (8).

Although water buffalo are particularly well-suited to rivers and marshy areas in humid tropical regions, they are kept as livestock on all inhabited continents. Not only are they used to reclaim farmland, but their yield of milk, leather, and meat is of economic importance (9–13). Animals infected with *Brucella* can spread it to humans through unpasteurized milk, meat, and animal by-products (14–17). Human brucellosis may result in fever, severe arthritis, and infertility; unfortunately, through misdiagnosis and mistreatment, it often becomes chronic. Repeated episodes may cause a serious economic burden to the family and nation through the loss of working days (18, 19). By developing effective vaccination plans and control strategies, some high-income countries have effectively controlled or even eliminated bovine brucellosis (20, 21); however, each year, some 500,000 persons continue to be infected, mostly in developing countries such as the Middle East and Southeast Asia (22–29). Despite the fact that Office International Des Epizooties (OIE) has proposed control measures for *Brucella* farms since 2010 (30), brucellosis infection has improved and the incidence of brucellosis in animals and humans has shown a significant upward trend in recent years.

As far as we know, no research has previously been done on the overall seroprevalence of buffalo brucellosis worldwide. Therefore, we conducted a systematic review and meta-analysis to analyze the total seroprevalence of buffalo brucellosis and assess the risk factors for the disease.

## Materials and Methods

### Search Strategy

We searched six databases for all publications on buffalo brucellosis: PubMed, Science Direct, Springer, China National Knowledge Infrastructure (CNKI), Wanfang Database (Wan Fang), and VIP Chinese Journal Databases (VIP). We searched for all studies between 1985 and May 16, 2020. In this study, titles and articles were searched using corresponding nouns in English or Chinese.

We searched using the Medical Subject Headings (MeSH) term “brucellosis” and related terms “Brucelloses,” “Malta Fever,” “Fever, Malta,” “Gibraltar Fever,” “Fever, Gibraltar,” “Rock Fever,” “Fever, Rock,” “Cyprus Fever,” “Fever,” “Cyprus,” “*Brucella* Infection,” “*Brucella* Infections,” “Infection, *Brucella*,” “Undulant Fever,” “Fever,” “Undulant,” “Brucellosis,” “Pulmonary,” “Brucelloses,” “Pulmonary,” and “Pulmonary Brucelloses.” We thus built Search Formula A: (((((((((((((((((“Brucellosis” [Mesh]) OR (Brucelloses)) OR (Malta Fever)) OR (Fever, Malta)) OR (Gibraltar Fever)) OR (Fever, Gibraltar)) OR (Rock Fever)) OR (Fever, Rock)) OR (Cyprus Fever)) OR (Fever, Cyprus)) OR (*Brucella* Infection)) OR (*Brucella* Infections)) OR (Infection, *Brucella*)) OR (Undulant Fever)) OR (Fever, Undulant)) OR (Brucellosis, Pulmonary)) OR (Brucelloses, Pulmonary)) OR (Pulmonary Brucelloses).

We then used the MeSH term “Buffalo” and its entries “Buffaloes,” “Syncerus,” “Water Buffaloes,” “Water Buffalo,” “Buffalo, Water,” and “Bubalus” to construct the search Formula B: ((((((“Buffaloes”[Mesh]) OR (Buffalo)) OR (Syncerus)) OR (Water Buffaloes)) OR (Water Buffalo)) OR (Buffalo, Water)) OR (Bubalus).

Finally, we used the logical “AND” of the two formulas to identify items satisfying both Search Formula A AND Search Formula B: ((((((((((((((((((“Brucellosis”[Mesh]) OR (Brucelloses)) OR (Malta Fever)) OR (Fever, Malta)) OR (Gibraltar Fever)) OR (Fever, Gibraltar)) OR (Rock Fever)) OR (Fever, Rock)) OR (Cyprus Fever)) OR (Fever, Cyprus)) OR (*Brucella* Infection)) OR (*Brucella* Infections)) OR (Infection, *Brucella*)) OR (Undulant Fever)) OR (Fever, Undulant)) OR (Brucellosis, Pulmonary)) OR (Brucelloses, Pulmonary)) OR (Pulmonary Brucelloses)) AND (((((((“Buffaloes”[Mesh]) OR (Buffalo)) OR (Syncerus)) OR (Water Buffaloes)) OR (Water Buffalo)) OR (Buffalo, Water)) OR (Bubalus)).

In ScienceDirect, we used the themes “Brucellosis” and “Buffaloes” and “seroprevalence” to search. In SpringerLink, we used the themes “Brucellosis” and “Buffaloes” and “seroprevalence” and “Buffaloes” to search. In Wanfang, we used the themes “Buffalo” and “*Brucella*” or “Buffalo” and “*Brucella*” to search. We used the themes “Buffalo” (in Chinese) and “Brucellosis” (in Chinese) in the CNKI database. In VIP, we used the themes or keywords “Buffalo” (in Chinese) and “*Brucella*” (in Chinese) or “Buffalo” (in Chinese) and “*Brucella*” (in Chinese); article types were limited to papers, either in journals or elsewhere, and collections.

In the three Chinese databases, i.e., CNKI, Wanfang, and VIP, the same search strategy for document retrieval was also used.

Studies were eligible if they satisfied all of the following inclusion criteria:

- The subjects of the research must be buffalo.
- The study's aim must be to investigate the seroprevalence of Buffalo brucellosis.
- Data must include the number of buffalo examined and the number of brucellosis-positive buffalo.
- The study must be published in Chinese or English.

Articles meeting any of the following exclusion criteria were excluded:

- host other than buffalo;
- review article;
- reviews or no data in studies;
- DOI does not match the article; and
- test method other than serology.

### Data Extraction and Quality Assessment

We collected data using standardized forms (31). The data records were as follows: country, region, environment, sampling year, method, breeding mode, gender, age group, national per capita income level, sample collection, health status, and whether the herd had contact with wild animals. We classified buffalo aged <1 year as suckling, buffalo aged 1 and 3 years as juvenile, and buffalo aged ≥3 years as adult. Articles were sorted according to region, sample classification (serum or milk), detection method, income level, gender, living environment, and age group of buffalo.

We assessed the quality of the included studies on a scale of 0–5 points, where 1 point was awarded for each of the following items: sampling time, clear detection method, detailed sampling method, random sampling, and analysis of four or more subgroups. According to this standard, we categorized studies by their total score as high (4–5 points), medium (2, 3), or low (0–1) in quality.

### Statistical Analysis

The “meta” package in R software (version 3.5.2) was used to obtain pooled prevalence rates (32). The degree of heterogeneity between the included studies, assessed using the χ^2^-based *Q*-test and *I*^2^, was used as the basis for selecting the effect model. Heterogeneity was considered insignificant when *P* > 0.1 and *I*^2^ < 50%. A fixed-effects model was used when *I*^2^ < 50%, *P* < 0.05, and a random-effects model was chosen when *I*^2^ > 50%, *P* < 0.05. Forest plots were used to visualize the statistical results of the meta-analysis (33, 34).

We used funnel plots, Egger's test, sensitivity analysis, and trim-and-fill analysis to evaluate the bias and stability of the selected articles (35). Publication bias was considered significant when Egger's test yielded *P* < 0.05 (36–38). A symmetric funnel plot exhibits no evidence of publication bias or heterogeneity (39). To estimate the impact of a single article on the results, we carried out a sensitivity analysis, repeating the meta-analysis with each article in turn deleted.

We used subgroup analysis to examine heterogeneity. The factors investigated included region (Europe vs. other continents), year of publication (publication year ≥ 2010 vs. < 2010), detection methods (serological vs. molecular biology methods), source of collected samples (serum vs. milk vs. other methods), farming mode (captive vs. stocking), living environment (wetlands vs. dry land), gender (female vs. male), age group (suckling vs. juvenile vs. adult), national income (high-income countries vs. middle- and low-income countries), whether the herd had contact with wild animals (with contact vs. without contact), and quality score (4–5 vs. 2–3 vs. 0–1).

## Results

### Search Results and Quality of the Eligible Studies

**Figure 1:**
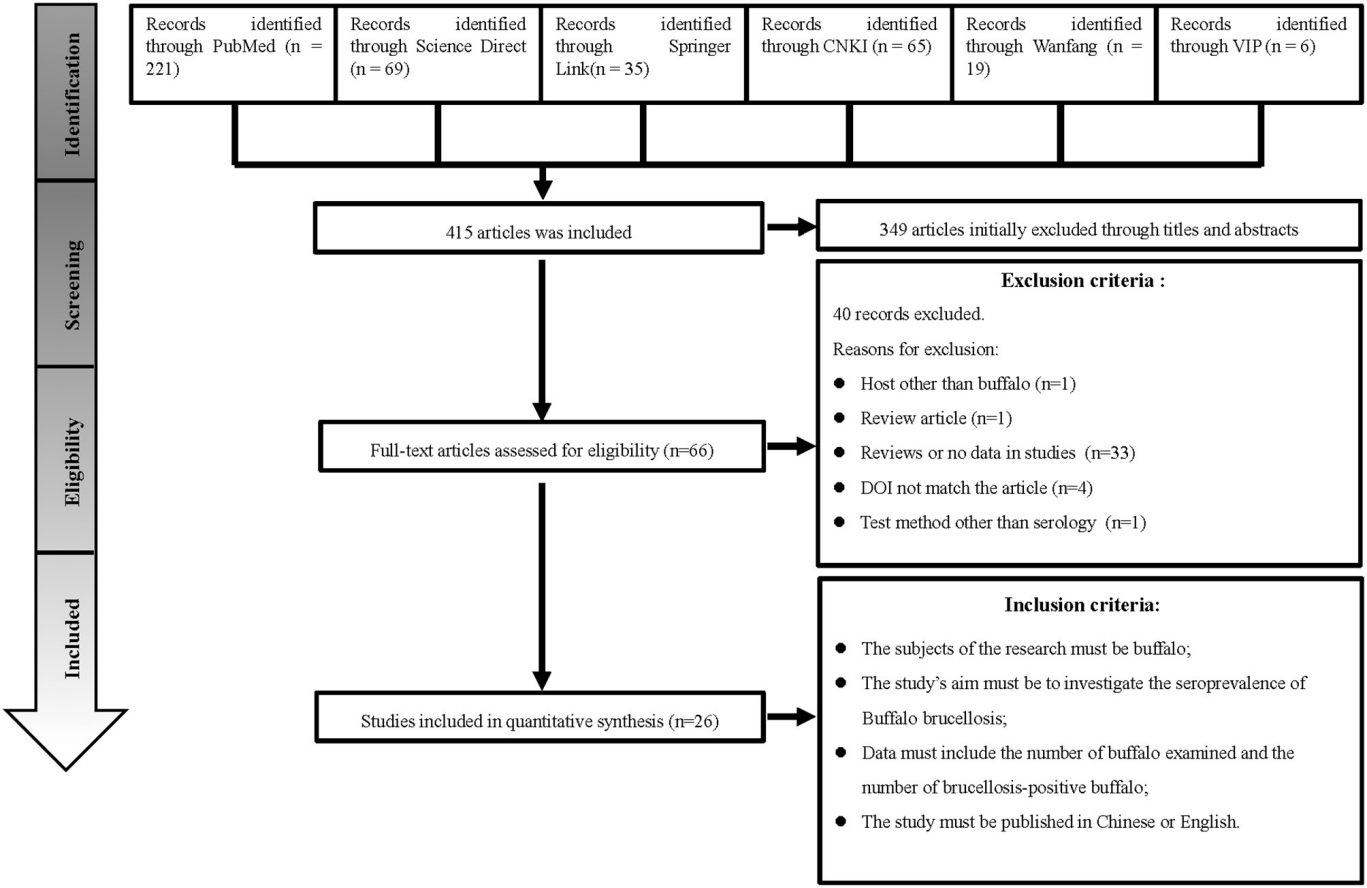
Flow diagram of literature search and selection.

We searched 415 studies from six databases. Based on the inclusion and exclusion rules, we analyzed 26 articles published between 1985 and 2020 (Figure 1). Ten papers were rated as high quality (4–5 points), and 16 papers were rated as medium quality (0–3 points); none of the included papers were rated as low quality (0–1).

### Results of Publication Bias

**Figure 2:**
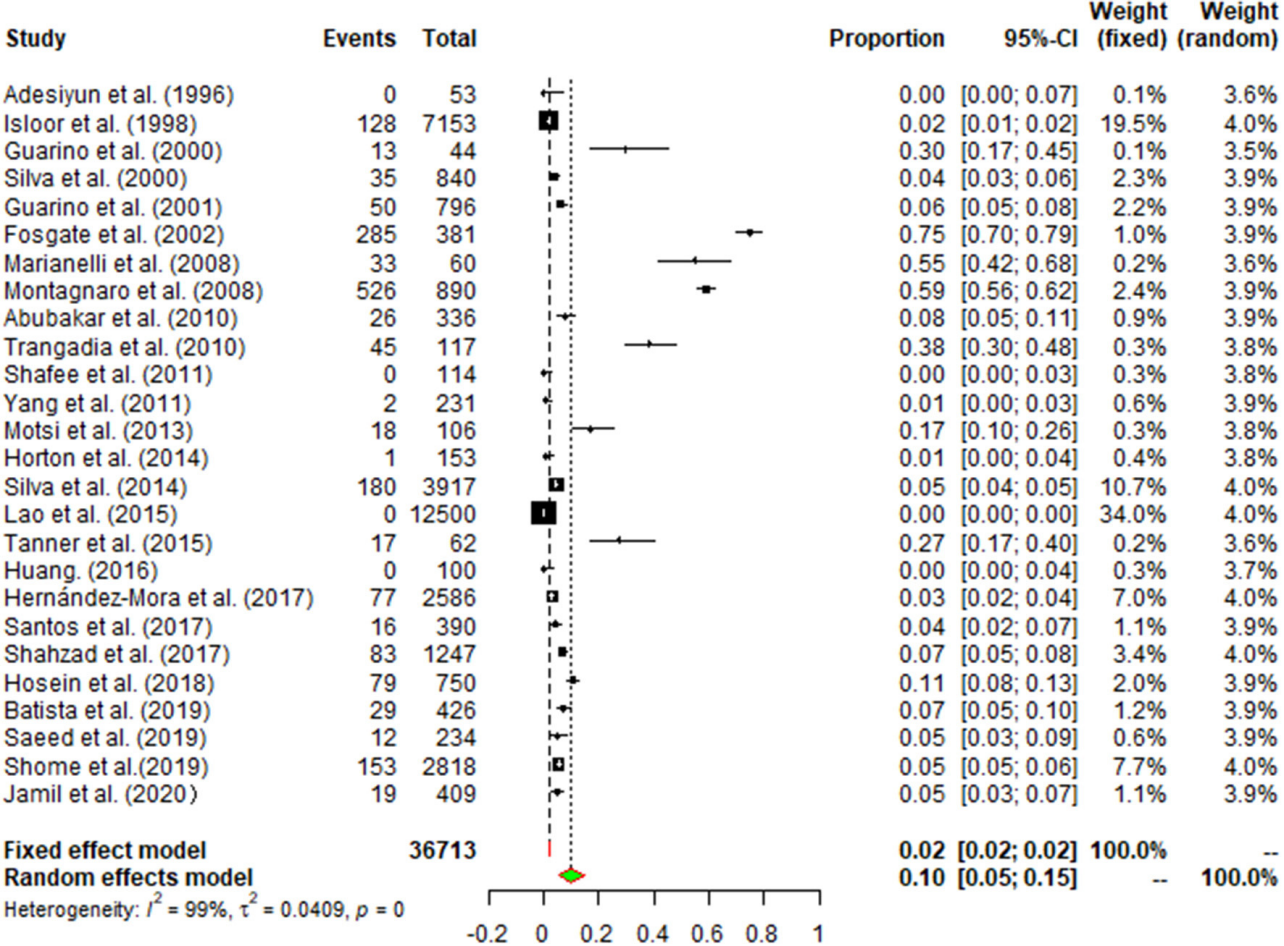
Forest plot of buffalo brucellosis among studies conducted anywhere in the world. The horizontal line represents the 95% CI; the diamond represents the pooled effect.

**Figure 3:**
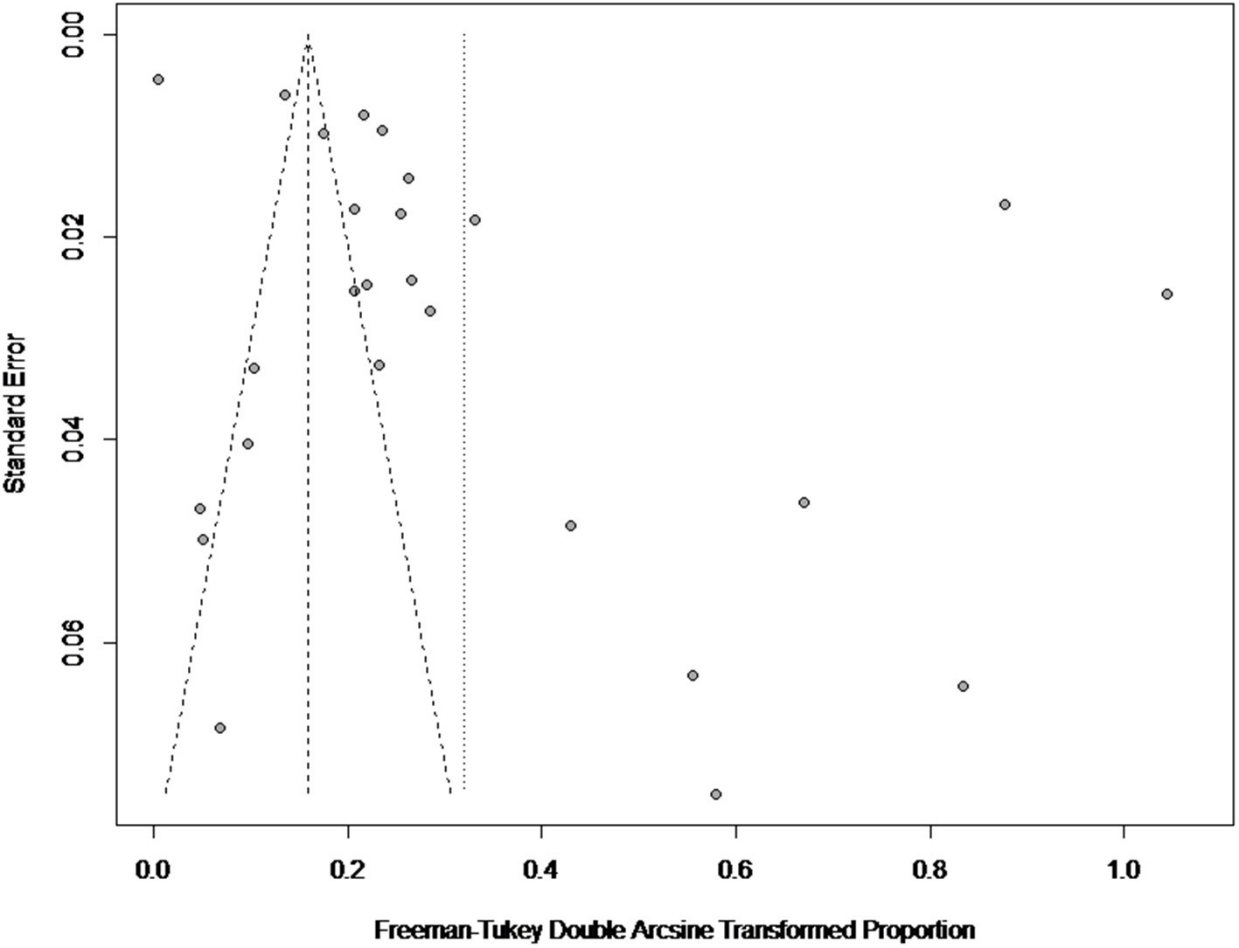
Funnel plot with pseudo 95% confidence interval for publication bias test.

**Figure 4:**
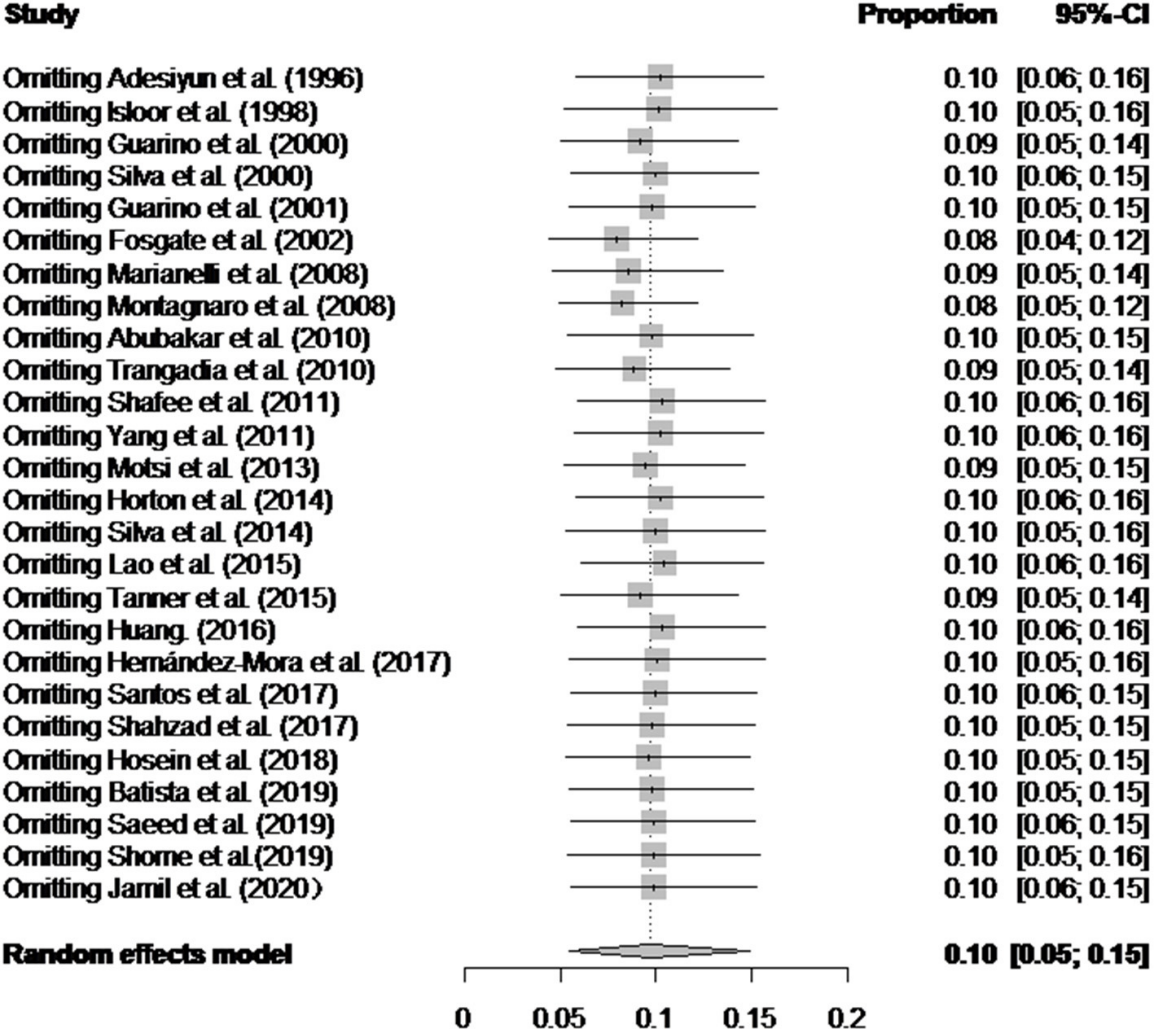
Sensitivity analysis. After one study at a time was removed, the remaining studies were recombined using a random-effects model to verify the impact of a single study on the overall results.

There was a high degree of heterogeneity between studies (*I*^2^ = 99.0%, *P* < 0.001) (Figure 2). The funnel plot shows evidence of publication bias in the selected articles (Figure 3), a result confirmed by Egger's test (*P* = 0.004) (Supplementary Figure 1). However, the test found that trimming did not add any research. The trim-and-fill analysis suggests that our own study is not publication bias (Supplementary Figure 2). Sensitivity analysis shows that after excluding a study, the results obtained are consistent with excluding the results obtained from other studies, which also suggests that our meta-analysis is trustworthy (Table 1 and Figure 4).

**Table 1:** Normal distribution test of the original rates and the different transformations of the original rates.

**Conversion form**	***W***	***P***
PRAW	0.69252	3.056e−06
PLN	NaN	NA
PLOGIT	NaN	NA
PAS	0.86086	0.001904
PFT	0.85362	0.001359

*PRAW, original rate; PLN, logarithmic conversion; PLOGIT, logit transformation; PAS, arcsine transformation; PFT, double-arcsine transformation; NaN, meaningless number; NA, missing data*.

### Meta-Analysis of Buffalo *Brucella* Seroprevalence in Buffalo Worldwide

The pooled seroprevalence of buffalo brucellosis was (9.7%) (95% CI: 5.5–14.9), based on data from 36,713 buffalo in selected articles (Table 2). In general, the data on seroprevalence mostly show a skewed distribution. The total seroprevalence of brucellosis in buffalo varies across different geographic regions (Tables 2, 3). Among regional groupings, Europe had the highest estimate of the midpoint, namely, 35.1% (95% CI: 4.8–74.6), and there was a statistically significant difference (*P* < 0.05) (Tables 2, 3). In the subgroup analysis by sampling year, the point estimates before 2010 (20.8%) (95% CI: 5.6–42.2) were higher than those after 2010 (4.2%) (95% CI: 1.8–7.5) (Table 2). The subgroup analysis by feeding mode found that the point estimate in stocked buffalo (11.5%) (95% CI: 3.6–23.0), was higher than that in captive buffalo (10.6%) (95% CI: 4.9–18.1); however, the difference was not statistically significant (*P* > 0.05). The subgroup analysis by farming mode found that the seroprevalence in artificially bred buffalo (10.7%) (95% CI: 6.6–15.7) was higher than that in intensively farmed buffalo (8.5%) (95% CI: 0.9–22.2). The subgroup analysis by farming environment found that the seroprevalence in buffalo living on dry land (6.4%) (95% CI: 2.0–12.9) was greater than that in buffalo living in wetlands (5.1%) (95% CI: 1.8–10.4) (*P* < 0.05). The point estimate in female buffalo (10.1%) (95% CI: 3.4–19.7) was higher than that in male buffalo (4.4%) (95% CI: 2.0–7.4); however, the difference was not statistically significant (*P* > 0.05) (Table 2). The point estimate of seroprevalence in high-income countries (32.8%) (95% CI: 7.5–65.2) was higher than that in middle-and low-income countries (Table 4). The age group with the highest estimated value was the lactating buffalo (26.9%) (95% CI: 1.8–66.5), but the difference between age groups was not significant (*P* > 0.05). The point estimate of *Brucella* in serum (10.3%) (95% CI: 5.9–15.8) was higher than that in milk (0%) (95% CI: 0–1.5). The subgroup analysis by study quality found the highest seroprevalence (10.8%) (95% CI: 5.5–17.6) for studies with a score of 4–5. The subgroup analysis by detection method found that the seroprevalence (27.3%) (95% CI: 0.7–70.8) detected by the complement fixation test (CFT) method was much higher than the rates detected by other methods.

**Table 2:** Studies included in the analysis.

**Study ID**	**Sampling time**	**Income level**	**Country**	**Detection methods**	**Positive samples/total samples**	**Quality score**	**Quality level**
**Asia**							
Isloor et al. (40)	1994–1997	Middle	India	SAT	128/7,153	4	High
Silva et al. (41)	1992.6–1995.8	Low	Sri Lanka	ELISA	35/840	4	High
Abubakar et al. (42)	UN	Low	Pakistan	RBPT	26/336	4	High
Trangadia et al. (43)	2007–2008	Middle	India	ELISA/RBPT	45/117	3	Middle
Muhammad et al. (44)	UN	Low	Pakistan	ELISA	0/114	2	Middle
Yang et al. (45)	2011.1–2011.2	Middle	China	Others	2/231	3	Middle
Lao et al. (46)	2012–2014	Middle	China	RBPT	0/12,500	3	Middle
Huang (47)	2014–2015	Middle	China	SAT	0/100	3	Middle
Ali et al. (48)	UN	Low	Pakistan	SAT	83/1,247	4	High
Saeed et al. (49)	UN	Low	Pakistan	RBT/PCR	12/234	4	High
Shome et al. (50)	UN	Middle	India	ELISA	153/2,818	4	High
Jamil et al. (51)	UN	Low	Pakistan	ELISA/RBPT	19/409	2	Middle
**North America**							
Adesiyun et al. (52)	1992.10–1995.10	High	Trinidad	SAT	0/53	3	Middle
Fosgate et al. (53)	1999.04–1999.06	High	Trinidad	SAT	285/381	4	High
Gabriela et al. (54)	2014–2016	Middle	Costa Rica	RBT/ELISA	77/2,586	3	Middle
**South America**							
Silva et al. (55)	2009–2011	Middle	Brazil	2-ME	180/3,917	4	High
Santos et al. (56)	UN	Middle	Brazil	2-ME/SAT	16/390	3	Middle
Batista et al. (57)	UN	Middle	Brazil	UN	29/426	2	Middle
**Europe**							
Guarino et al. (58)	UN	High	Italy	PCR/CTF/ELISA	13/44	2	Middle
Guarino et al. (59)	UN	High	Italy	ELISA	50/796	2	Middle
Marianelli et al. (60)	UN	High	Italy	ELISA/PCR	33/60	2	Middle
Montagnaro et al. (61)	UN	High	Italy	RBT/CFT	526/890	2	Middle
**Africa**							
Motsi et al. (62)	2009–2012	Low	Zimbabwe	RBT/CFT	18/106	5	High
Horton et al. (63)	2009.07	Low	Egypt	SAT	1/153	3	Middle
Tanner et al. (64)	UN	Middle	South Africa	RBT/ELISA	17/62	3	Middle
Hosein et al. (65)	2016.09–2017.04	Low	Egypt	CFT	79/750	4	High

*UN, unclear; ND, no data*.

**Table 3:** Pooled prevalence of *Brucella* in buffalo worldwide.

		**No. studies**	**No. tested**	**No. positive**	% (95% CI^*^)	**Heterogeneity**				**Univariate meta-regression**
						**χ^2^**	***P*-value**	***I*^2^ (%)**	***P*-value**	**Coefficient (95% CI)**
Region^*^										
	Asia	12	26,099	503	3.9% (1.5–7.2)	1,105.92	<0.01	99.0%		
	North America	3	3,020	362	17.2% (0–78.5)	1,017.03	<0.01	99.8%		
	South America	3	4,733	225	5.0% (3.7–6.3)	4.08	0.13	51.0%		
	Europe	4	1,790	622	35.1% (4.8–74.6)	666.23	<0.01	99.5%	0.0002	0.368 (0.176 to 0.560)
	Africa	4	1,071	115	11.3% (3.3–22.9)	49.66	<0.01	94.0%		
**Sampling years**										
	After 2010	16	25,890	685	4.2% (1.8–7.5)	1,395.04	<0.01	98.9%		
	Before 2010	10	10,487	1,116	20.8% (5.6–42.2)	2,955.04	0	99.7%	0.0026	0.262 (0.091 to 0.433)
**Quality level**										
	4–5	10	17,782	999	10.8% (5.5–17.6)	1,317.67	<0.01	99.3%	0.7643	0.027 (−0.148 to 0.201)
	2–3	16	18,931	828	9.1% (2.6–18.7)	3,202.11	0	99.5%		
	0–1	0	0	0	ND	ND	ND	ND	ND	ND
Method^*^										
	RBPT	6	16,010	113	6.5% (2.1–13.0)	409.32	<0.01	98.8%		
	SAT	6	9,087	497	7.3% (0–25.1)	1,243.59	<0.01	99.6%		
	ELISA	11	10,432	508	13.5% (8.5–19.4)	544.23	<0.01	98.2%		
	CFT	2	794	101	27.3% (0.7–70.8)	34.64	<0.01	97.1%	0.0997	0.209 (−0.040 to 0.458)
**Reproductive health**										
	Reproductive issues	2	1,607	101	4.8% (3.6–6.0)	0.30	0.58	0.0%		
	Health	2	2,736	108	4.8% (2.1–8.7)	8.60	<0.01	88.4%	0.5150	−0.039 (−0.157 to 0.079)
**Feeding mode**										
	Captive	13	25,833	829	10.6% (4.9–18.1)	2,641.95	0	99.5%		
	Stock	10	7,857	931	11.5% (3.6–23.0)	1,469.46	<0.01	99.4%	0.8654	0.015 (−0.159 to 0.189)
**Farming mode**										
	Intensification	10	15,824	509	8.5% (0.9–22.2)	2,246.06	0.00	99.6%		
	Artificial	8	12,998	526	10.7% (6.6–15.7)	320.63	<0.01	97.8%	0.5670	0.059 (−0.142 to 0.259)
**Environment**										
	Wetland	8	18,016	234	5.1% (1.8–10.4)	678.65	<0.01	99.0%		
	Dryland	6	3,796	239	6.4% (2.0–12.9)	120.24	<0.01	95.8%	0.7100	0.028 (−0.121 to 0.178)
**Sex**										
	Male	2	266	13	4.4% (2.0–7.4)	0.45	0.50	0.0%		
	Female	3	4,183	214	10.1% (3.4–19.7)	27.63	<0.01	92.8%	0.4600	0.080 (−0.132 to 0.292)
Age of buffalo^*^										
	Lactating buffalo	3	2,378	447	26.9% (1.8–66.5)	742.12	<0.01	99.7%	0.2619	0.247 (−0.185 to 0.680)
	Juvenile buffalo	2	3,934	181	3.4% (2.8–4.1)	0.37	0.54	0.0%		
	Adult buffalo	2	477	37	10.8% (1.1–28.0)	14.73	<0.01	93.2%		
**Income level**										
	High	6	2,224	907	32.8% (7.5–65.2)	1,000.03	<0.01	99.5%	<0.0001	0.386 (0.244 to 0.528)
	Middle	11	30,300	647	5.0% (2.3–8.6)	1,225.49	<0.01	99.2%		
	Low	9	4,189	273	5.3% (3.2–7.9)	76.94	<0.01	89.6%		
**Country**										
	China	3	12,831	2	0.5% (0–1.2)	9.67	<0.01	79.3%		
	Pakistan	5	2,340	218	4.6% (2.5–7.2)	22.64	<0.01	82.3%		
	Trinidad	2	434	285	27.8% (0–99.9)	178.75	<0.01	99.4%		
	Brazil	3	4,733	225	5.0% (3.7–6.3)	4.08	0.13	51.0%		
	India	3	10,088	326	10.4% (3.9–19.3)	202.04	<0.01	99.0%		
	Italy	4	1,790	622	35.1% (3.8–74.6)	666.23	<0.01	99.5%		
	Costa Rica	1	2,586	77	3.0% (3.7–7.6)	0.00	ND^*^	ND		
	Egypt	2	903	80	4.5% (0–18.5)	27.90	<0.01	96.4%		
	Zimbabwe	1	106	18	17.0% (10.4–24.8)	0.00	ND	ND		
	Sri Lanka	1	840	35	4.2% (2.9–5.6)	0.00	ND	ND		
	Africa	1	62	17	27.4% (17.0–39.3)	0.00	ND	ND		
**Sample**										
	Serum	25	36,599	1,827	10.3% (5.9–15.8)	4,942.66	0.00	99.5%	0.1798	0.284 (−0.131 to 0.699)
	Milk	1	114	0	0% (0–1.5)	0.00	ND^*^	ND^*^		
Wild^*^										
	No	2	206	7	2.1% (0–12.9)	9.83	<0.01	89.8%		
	Yes	2	426	40	11.1% (3.1–22.9)	8.27	<0.01	87.9%	0.1583	0.185 (−0.072 to 0.443)
Total		26	36,713	1,827	9.7% (5.5–14.9)	4,948.43	0.00	99.5%		

CI*: Confidence interval.

Region*: Asia: China, Pakistan, India, and Sri Lanka; North America: Trinidad and Costa Rica; South America: Brazil; Europe: Italy; Africa: Egypt, Zimbabwe, and South Africa.

Method*: CFT, Complement fixation test; ELISA, Enzyme-linked immunosorbent assay; RBPT, Rose Bengal plate test; SAT, Serum agglutination test.

Age of buffalo*: Lactating buffalo: >1 year; Juvenile buffalo: 1–3 years; Adult buffalo: >3 years.

Others*: Aborted fetuses and vaginal swabs.

ND*: no data.

Wild*: Contact with wild animals.

**Table 4:** Estimation of the seroprevalence of *Brucella* in buffalo in various countries.

**Countries**	**No. studies**	**Region**	**No. tested**	**No. positive**	**% Prevalence**	**% (95% CI)**
China	3	Asia	12,831	2	0.05%	0–1.2
Pakistan	5	Asia	2,340	218	4.6%	2.5–7.2
Trinidad	2	North America	434	285	27.8%	0–99.9
Brazil	3	South America	4,733	225	5.0%	3.7–6.3
India	3	Asia	10,088	326	10.4%	3.9–19.3
Italy	4	Europe	1,790	622	35.1%	4.8–74.6
Costa Rica	1	North America	2,586	77	3.0%	2.4–3.7
Egypt	2	Africa	903	80	4.5%	0–18.5
Zimbabwe	1	Africa	106	18	17.0%	10.4–24.8
Sri Lanka	1	Asia	840	35	4.2%	2.9–5.6
South Africa	1	Africa	62	17	27.4%	2.9–5.6
Total	26		37,875	1,905	9.6%	5.5–14.6

### Results of Sensitivity Analysis

Italy's point estimate (35.1%) (95% CI: 3.8–74.6) was the highest for any country; however, according to Table 2, the sampling time was unclear for all four of the studies from Italy, all of which were published before 2010. Guarino found a seropositive rate of 10% in the detection of *Brucella* in Italy (59); Montagnaro tested for *Brucella* in Campania in southern Italy with a seropositive rate of 59.1% (60). Therefore, it is unclear whether the very high value reported for the country is still representative.

Results of univariate multiple regression are shown for variables including region, country, sampling year (1985–2020), presence or absence of problems with reproduction, reproduction method, living environment, income, and contact with wild animals. These groups may be the main source of heterogeneity in the meta-analysis (Table 2).

## Discussion

Brucellosis is a major epidemic disease in cattle-raising areas around the world, which can cause buffalo abortion and cause economic losses (66). In addition, due to the close relationship between buffalo and humans, brucellosis can be transmitted to humans (67). This study is the world's first meta-analysis of the seroprevalence of buffalo brucellosis. In this study, we searched six databases and found a total of 26 related articles, which contained qualifying data.

Before 2010, the global infection rate was much higher than after 2010. We speculate that the global reduction in infection rate after 2010 is attributable to on-site control measures for *Brucella* that the World Organization for Animal Health (OIE) proposed in the chapter on brucellosis in its 2010–2011 terrestrial animal health regulations. Greater attention to brucellosis has led to improved buffalo breeding technology in countries around the world. The control of diseases in developing countries has gradually matured, and certain diseases have been well-controlled. However, countries whose preventive measures are not yet adequate need to be vigilant and, when formulating and applying animal food safety standards, should prioritize pathogens that affect human health. A research focus on developing countries and countries with transitional economies would support the aim of reducing global poverty (58).

Our research shows that the seroprevalence of buffalo brucellosis is different for different regions and different periods. We believe that many factors contribute to different epidemics in different regions. First of all, there are significant regional differences in breeding scale, breeding technology, and on-site sanitation conditions. In some areas, especially underdeveloped areas, the proportion of free-range buffalo is relatively large (68). Secondly, buffalo often inhabit muddy or flooded areas, where *Brucella* can survive longer (69); therefore, the point estimate is higher in areas with tropical climates. Finally, we should note that our systematic search found few studies on buffalo brucellosis seroprevalence relative to the number of countries where buffalo are raised, and in some countries, only one or two studies have been analyzed. Therefore, our conclusions are necessarily limited and should be applied with caution in consideration of local conditions that may affect seroprevalence. Regional diversity in the infection of buffalo by *Brucella* needs further study.

The seroprevalence of buffalo brucellosis may be related to the age of the buffalo. Studies have pointed out that *Brucella* exposure may occur in the first year after weaning. For animals older than 1 year, the average probability of seropositivity decreases with age (70, 71). We attribute this trend to incomplete development of immune function during the suckling period, with the result that resistance to diseases becomes more sufficient only as the animal continues to mature. From the results of gender subgroups, the seroprevalence of females is higher than that of males, but the difference is not significant. As the number of male diseased buffalo in our included articles was only 266, this is not sufficient to prove that female buffalo are more prevalent than males. Female animals can transmit infection to other livestock through skin and mucous membranes (72). At the same time, most farms have far more female animals than male animals, the young animals can be infected by contact with the secretions and excrement of the mother animals; male animals may be infected through sexual contact with the mother animals. Our limited data on fetal miscarriage are treated separately, and since our search found only one article on fetal miscarriage, a pooled analysis was not possible. We recommend that future epidemiological research into brucellosis examine the positive rate of aborted fetuses.

Our results found that from the perspective of national income and farming methods, in countries with a high per-capita income and middle-income countries, such as Italy and China, respectively, buffalo breeding adopts intensive breeding strategies; the infection rate of buffalo brucellosis is high in countries with low per-capita income such as Pakistan and India [see, e.g., (57)]. Where artificial breeding is the main strategy for buffalo, the infection rate of buffalo brucellosis is higher. The potential explanation for this model may be that low- and middle-income levels are represented in this analysis by Pakistan, South Africa, and China, which are characterized by developing agriculture, so the trend may be generalized to other countries with similar per-capita income. The results of a previous study showed that the burden of buffalo brucellosis in low-income countries was lighter than that in higher-income countries, which also supports our results (73). As human settlements have expanded, they have encroached upon the habitat of wild animals, thereby increasing the possibility of contact between people and wild animals (74, 75). This may contribute to increased chances of brucellosis infection, first in livestock and consequently in humans consuming infected food. Therefore, it is necessary to conduct regular inspections and to enhance breeders' awareness because their perceptions of brucellosis may otherwise interfere with taking effective preventive measures (68). Once an inspection finds a positive result, the sick cattle must be dealt with promptly by processing methods that include culling and burying (76). Treatment should focus on incineration and landfill as much as possible (30). At the same time, most buffalo live in humid environments, tropical climates or tropical rain forest climates are mostly wetlands, and humid environments are more susceptible to buffalo infections than dry environments (30). However, data show that the seropositive rate of brucellosis in buffalo living on dry land was higher than that in those living in wetlands. This may be due to the fact that most of the dry land in the extracted data is in low- and middle-income countries and to the herders' lack of awareness of brucellosis prevention.

Based on the classification of the samples collected from buffalo, *Brucella* is more likely to be detected in serum than in other sources, possibly because most tests use blood as a sample. Milk with mastitis and other breast diseases is not suitable for testing and is prone to false positives (77), which affects the detection result. The Rose Bengal plate test (RBPT) mainly detects IgG antibodies (77, 78). This simple method of qualitative analysis can complete preliminary screening quickly (79). However, due to the interference of non-specific antibodies, non-specific agglutination sometimes occurs. The serum agglutination test (SAT) is a serological test that detects the agglutination of an antigen when it binds to the corresponding antibody, so that results can be directly observed in the test tube by the naked eye. However, not only does the test take 18–24 h to complete, but it also requires a large amount of *Brucella* agglutination antigen and diseased livestock serum, and a standard turbidimetric tube needs to be made during the test, and the results must be compared one by one, which is time-consuming and laborious (80). The enzyme-linked immunosorbent assay (ELISA) is divided into I-ELISA and C-ELISA (81). ELISA refers to a qualitative and quantitative detection method in which soluble antigens or antibodies are bound to solid-phase carriers such as polystyrene, and the specific binding of antigen and antibody is used to carry out the immune response (82). The CFT method works mainly through the combination of the *Brucella* complement binding test antigen and the antibody in the serum to be tested, thereby forming a complex (83), and the complex is combined with the complement, no longer causing hemolysis of red blood cells in the indicator system, and the result is positive; otherwise, it is negative. From the point of view of detection methods, the CFT method for detection of brucellosis detected the highest positive rate. As a commonly used detection technique in serology, CFT has the characteristics of strong specificity, high sensitivity, and easy promotion compared with other serological detection methods such as the SAT, so the OIE has approved it for serological diagnosis of brucellosis.

Our research has some limitations. First, we tried to identify buffalo brucellosis infection by using several different MeSH terms in all studies in the selected database. Second, we found few studies on buffalo brucellosis infection in countries around the world, sampling locations are concentrated, and the sample size included in the study in some countries is very small. Therefore, it is difficult to objectively determine the true incidence of buffalo brucellosis in any specific locality. Third, limitation of the language of publication to Chinese or English may have led to the omission of relevant research in other languages, so the included studies may not be fully representative of certain regions. Fourth, some of the included studies did not indicate whether sampling was random, which may lead to heterogeneity. Finally, because some included papers have not reported the sample area and number of sample years, we did not conduct a joint test of the sampling area and year for each risk factor, which may lead to instability.

Despite notable improvements since 2010, buffalo brucellosis has remained a high-risk factor for human infection even in recent years. To improve public health, it is necessary to take appropriate management and monitoring measures for brucellosis infection, so it is strongly recommended to enhance herders' awareness of this disease and reduce errors in the feeding process. Better monitoring and control of the disease in animals can keep meat and dairy products from causing brucellosis in humans.

## Data Availability Statement

The original contributions presented in the study are included in the article/Supplementary Material, further inquiries can be directed to the corresponding author/s.

## Author Contributions

J-ML and RD: idea contributions. RD, XL, and YZ: funding. Z-YC, Y-HS, YY, and B-YM: data extraction. BZ: database establishment. QW and XL: data analysis. J-FS: writing—original draft. Q-LG and YZ: writing—review and editing. All authors contributed to manuscript editing and approved the final manuscript.

## Conflict of Interest

The authors declare that the research was conducted in the absence of any commercial or financial relationships that could be construed as a potential conflict of interest.

## Supplementary Material

Supplementary Figure 1 Egger's test for publication bias.

Supplementary Figure 2 Funnel plot with trim and fill analysis for the publication bias test.

## Abbreviations

OIE: World Organization for Animal Health
CFT: Complement fixation test
ELISA: Enzyme-Linked Immunosorbent Assay
RBPT: Rose Bengal plate test
SAT: Serum agglutination test.
