# Supplementary figures and images for: Seroprevalence of Brucellosis in Buffalo Worldwide and Associated Risk Factors: A Systematic Review and Meta-Analysis
Source: Front Vet Sci. 2021 Jun 4;8:649252. doi: 10.3389/fvets.2021.649252 (PMC8213021; doi:10.3389/fvets.2021.649252)

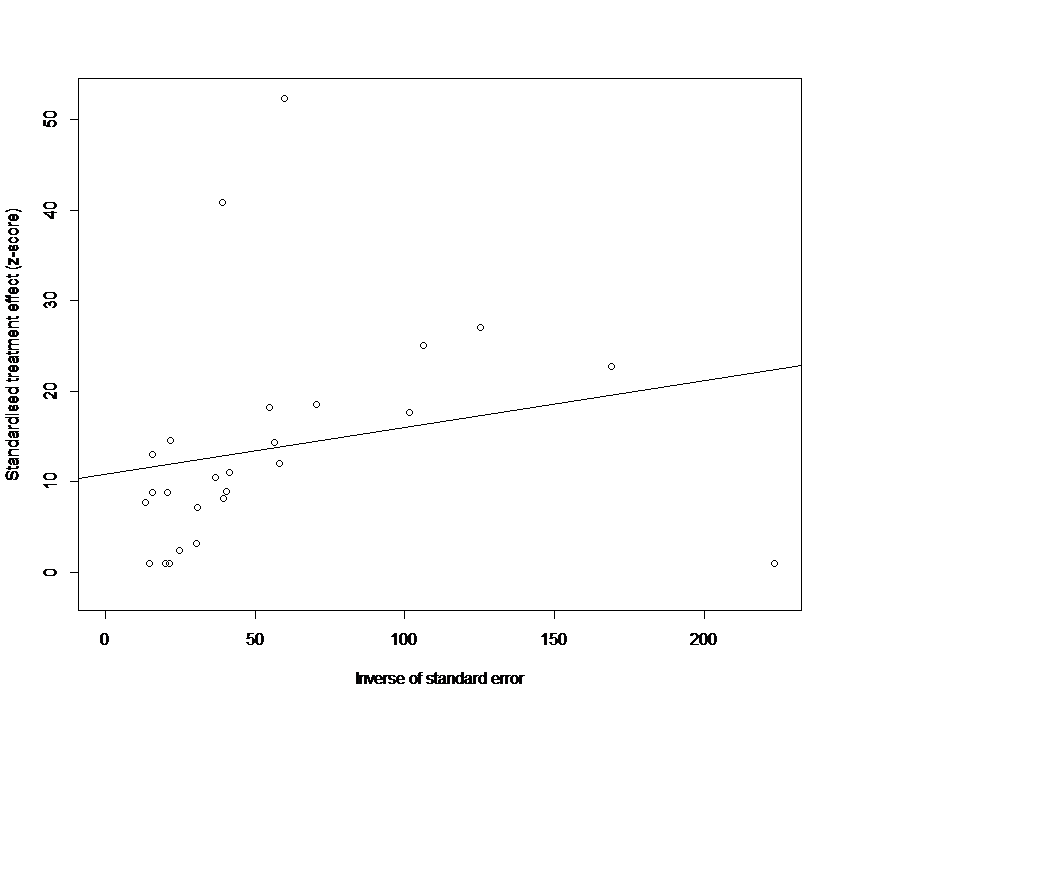

Supplement: Supplementary Figure 1 — Egger's test for publication bias. [file Image_1.TIF]

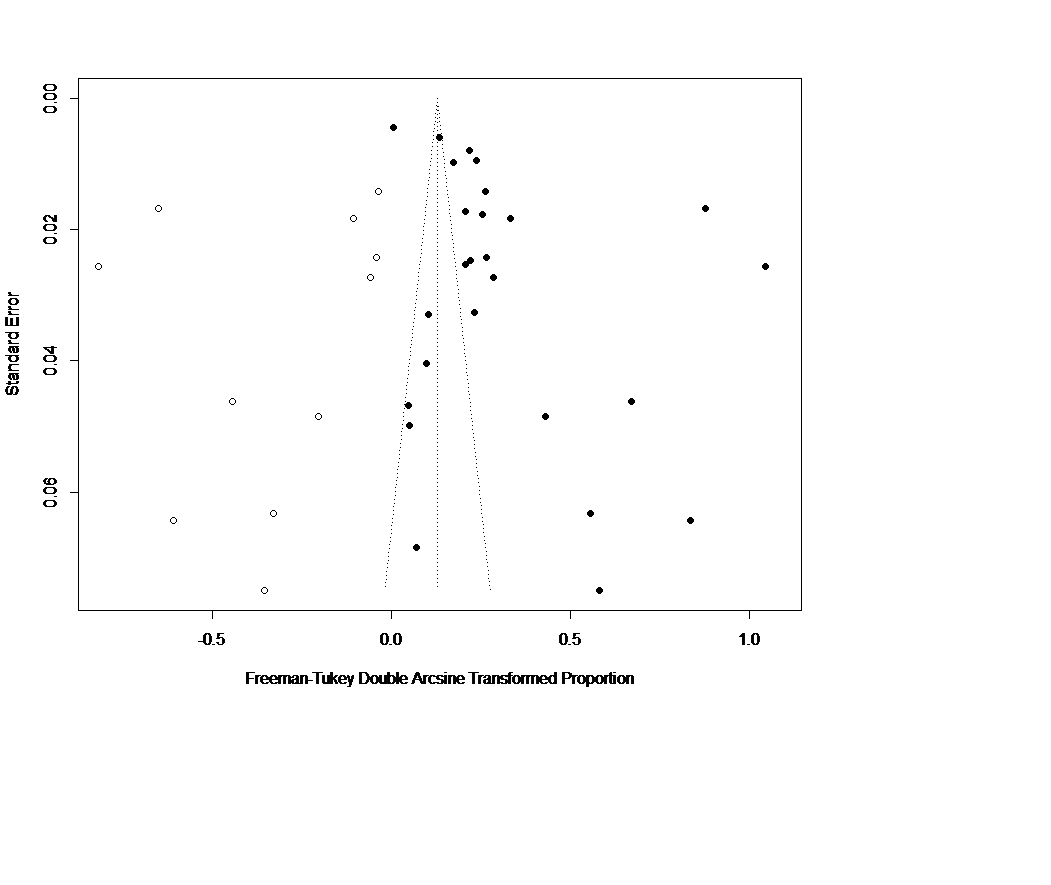

Supplement: Supplementary Figure 2 — Funnel plot with trim and fill analysis for the publication bias test. [file Image_2.TIF]
